# A gene for plant protection: expression of a bean polygalacturonase inhibitor in tobacco confers a strong resistance against *Rhizoctonia solani* and two oomycetes

**DOI:** 10.3389/fpls.2012.00268

**Published:** 2012-12-05

**Authors:** Orlando Borras-Hidalgo, Claudio Caprari, Ingrid Hernandez-Estevez, Giulia De Lorenzo, Felice Cervone

**Affiliations:** ^1^Center of Biotechnology and Genetic EngineeringLa Habana, Cuba; ^2^Department of Bioscience and Territory, University of MolisePesche (IS), Italy; ^3^Department of Biology and Biotechnology, Sapienza University of RomeRome, Italy

**Keywords:** disease resistance, polygalacturonase inhibitor, oomycetes, plant protection

## Abstract

We have tested whether a gene encoding a polygalacturonase-inhibiting protein (PGIP) protects tobacco against a fungal pathogen (*Rhizoctonia solani*) and two oomycetes (*Phytophthora parasitica* var. *nicotianae* and *Peronospora hyoscyami* f. sp.* tabacina*). The trials were performed in greenhouse conditions for *R. solani* and *P. parasitica* and in the field for *P. hyoscyami*. Our results show that expression of PGIP is a powerful way of engineering a broad-spectrum disease resistance.

## INTRODUCTION

Plants that in nature are exposed to biotic stresses often resist to pathogen infection by rapidly activating the innate immune system. An efficient activation of the resistance responses relies on the prompt perception/transduction of signal molecules that are common to many classes of pathogens (pathogen-associated molecular patterns, PAMPs) and are recognized by germ line-encoded pattern recognition receptors (PRRs). Resistant responses are also triggered by race-specific molecules (effectors, Avr products) recognized by the so-called Resistance (R) proteins that are present in specific cultivars of several but not all crop plants (Jones and Dangl, 2006). R proteins have been widely used in breeding programs as well as for genetic transformation to protect plants against specific pathogen genotypes. Usually R-mediated resistance does not last for a long time since pathogens continually evolve more aggressive genotypes. More recently it has been proposed that a PRR-mediated recognition of PAMPs may be utilized to confer to transgenic plants a larger spectrum of disease resistance. Indeed, the Arabidopsis EFR that recognizes the bacterial elongation factor EF-Tu has been shown to confer resistance against several bacteria when transferred into Solanaceae plants (Lacombe et al., 2010). It has also been proven that chimeric PRRs may be used to engineer resistance against both bacteria or fungi (Brutus et al., 2010; De Lorenzo et al., 2011). The combination in a single plant of different PRRs as well as of chimeric PRRs that recognize several non-self structures likely represents the best way of constructing broad-spectrum and long lasting disease resistances.

The plant cell walls constitute the first line of defense against microbes. The majority of pathogenic microorganisms produce cell wall degrading enzymes (CWDEs) that are especially important for those pathogens that lack specialized penetration structures. Among the different CWDEs produced by fungi polygalacturonases (PGs) play a critical role since their action on pectin makes other cell wall components more accessible to other CWDEs (Lionetti et al., 2010). Consequently, as a strategy to optimize the action of CWDEs, PGs are often the first enzymes secreted by pathogens growing on the plant cell walls (De Lorenzo et al., 2001). PG-inhibiting proteins (PGIPs) are well-characterized proteins that recognize microbial and insect PGs and interfere with the plant cell wall degradation during pathogen attacks. These proteins are leucine-rich repeat (LRR) proteins like most of the R proteins and several PAMP receptors (Casasoli et al., 2009). They not only inhibit PGs and retard the hydrolysis of pectin but also favor the accumulation of oligogalacturonides (OGs), a class of damage-associated molecular patterns (DAMPs) that, like PAMPs, activate the plant innate immunity system (Brutus et al., 2010). The importance of PGIPs in resistance against the necrotrophic fungus *Botrytis cinerea* is well established: transgenic tomato and grapevine plants expressing a pear PGIP or transgenic tobacco and *Arabidopsis* plants expressing, respectively, bean or *Arabidopsis* PGIPs are more resistant to *Botrytis* infection in greenhouse experiments (Powell et al., 2000; Ferrari et al., 2003; Agüero et al., 2005; Manfredini et al., 2005).

Conversely, *Arabidopsis* plants expressing an antisense *pgip* gene are more susceptible to this fungus (Ferrari et al., 2006). In spite of the lower quantity of pectin in their cell wall, also monocots are protected by transgenic expression of a bean PGIP in greenhouse trials against fungi (*Fusarium graminearum* and *Bipolaris sorokiniana*; Janni et al., 2008; Ferrari et al., 2011). A negative case is represented by tomato transgenic plants expressing PvPGIP1. Due to the limited ability of PGIP1 to inhibit PGs from *F. oxysporum* f. sp. *lycopersici*, *B. cinerea*, and *Alternaria solani* the transgenic plants did not exhibit enhanced resistance against these fungi (Desiderio et al., 1997). Here, we have tested whether PvPGIP2 from *Phaseolus vulgaris *L. protects tobacco against an important fungal pathogen (*Rhizoctonia solani*) and two dangerous oomycetes (*Phytophthora parasitica* var. *nicotianae *and *Peronospora hyoscyami *f. sp. *tabacina*). Greenhouse conditions were tested for *R. solani* and *P. parasitica* var. *nicotianae* while field trials were carried out for *P. hyoscyami* f. sp. *tabacina*. We propose that the use of PGIP is a powerful way of engineering a broad-spectrum disease resistance.

## MATERIALS AND METHODS

### TRANSGENIC PLANTS

Two independent transgenic lines of *Nicotiana tabacum* cv. Petit Havana SR1 plants overexpressing PvPGIP2 of *P. vulgaris* (accession number P58822) have been used in this work and belong to the collection of plants previously characterized (Manfredini et al., 2005). The line indicated here as 2005 corresponds to the line 12.5 described in Manfredini et al. (2005). The normal phenotype of line 2.1 and its ability to revert the dwarf phenotype of transgenic tobacco plants expressing the *Aspergillus niger* PG II have been previously described (Capodicasa et al., 2004; Manfredini et al., 2005).

### DISEASE RESISTANCE ASSAY

#### Inoculation with R. solani

Transgenic and control tobacco plants (*N. tabacum *cv. Petit Havana SR1 provided by the Tobacco Research Institute, Cuba) were grown in 6-inch pots containing black turf and rice husk (4:1) and maintained in growth chambers at 23°C. An aggressive isolate belonging to anastomosis group *n*. 3 of *R. solani* (kindly provided by the Cuban Research Institute of Plant Health and characterized by sequencing the ITS region that matched the sequence of isolate AG3, GenBank accession number HQ241274.1) was used for inoculations. The isolate was grown on potato dextrose agar at room temperature (22–25°C) for 5 days. Colonized agar plugs were removed and transferred to 250-ml Erlenmeyer flasks containing autoclaved rice grains. The pathogen was allowed to colonize the rice grains for approximately 2 weeks at room temperature and the grains were used to inoculate tobacco. Two-week-old tobacco seedlings were inoculated with approximately six grains onto the surface of the soil according to Elliott et al. (2008). Mock-inoculated untransformed plants were used as controls. Typical symptoms caused by *R. solani* were monitored visually at 0, 1, 2, and 3 weeks post-inoculation (wpi). Growth of *R. solani* on tobacco was estimated by quantitative real-time reverse transcription PCR. The extent of colonization was determined by the ratio of transcripts of the constitutively expressed actin gene (measuring the fungal biomass) to the constitutively expressed tobacco 26S rRNA gene (measuring the plant biomass) shown on a linear scale. Amplification products were sequenced and confirmed to correspond to the *R. solani* actin and the tobacco rRNA transcripts. Disease incidence, which included the percentage of plants exhibiting seedling death and stem rot, was evaluated after 3 wpi according to Elliott et al. (2008). The PCR product generated was sequenced and confirmed the origin. For each time point, three root samples were taken from five plants and the experiment was repeated twice. An arcsine transformation was performed on all percent incidence data before statistical analysis in order to improve homogeneity of variance. Data were analyzed by analysis of variance or general linear model procedures of SAS (SAS Institute, Cary, NC, USA). Significant difference among mean values was determined by Fisher’s least significant difference mean separation at *P* = 0.05.

#### Inoculation with P. parasitica var. nicotianae

The pathogen *P. parasitica* var. *nicotianae* race 0 used in this study belongs to the Plant Health Institute in Havana and was isolated from naturally infected tobacco plants in Havana fields. The isolate was identified and classified through sequencing the ITS region that matched the sequence of the GenBank isolate with accession number DQ059571.1. The inoculum was prepared by sterilizing and infesting toothpicks with the test organism. Toothpicks were autoclaved in V8 juice for 15 min at 121°C, allowed to cool, placed on Petri plates filled with potato dextrose agar, and inoculated with a 5-mm plug from actively growing cultures of *P. parasitica* var. *nicotianae*. Plates were incubated at 27°C in the dark for 10–14 days, allowing the fungus to grow across the plates and into the media-impregnated toothpicks.

During the greenhouse evaluation the tobacco transgenic lines and wild type plants were inoculated by aseptically pushing the infested toothpicks into stems 2–3 cm above the soil line or into root systems near the base of the plant (Sullivan et al., 2005). Greenhouse temperature was ranging from 15 to 25°C during the tests. Uninfected toothpicks acted as controls. Each treatment had 50 plants and was replicated five times. Each transgenic line and wild type plant was kept in separate trays and placed in separate float baths to prevent cross contamination during the experiment. Development of stem lesions was evaluated using a linear scale of 1–10, where 1 was no disease and 10 was a dead plant according to Csinos (1999). The ratings were taken on stems at 10 days post-inoculation. An arcsine transformation was performed on all percent incidence data before statistical analysis in order to improve homogeneity of variance. Data were subjected to analysis of variance or general linear model procedures of SAS (SAS Institute, Cary, NC, USA). Significant difference among means was determined by Fisher’s least significant difference mean separation at *P* = 0.05.

#### Resistance to P. hyoscyami f. sp. tabacina

The *P. hyoscyami* f. sp. *tabacina* isolate belongs to the Plant Health Institute in Havana and was collected from a tobacco field near Havana, identified and classified through sequencing the ITS region that matched the sequence of the GenBank isolate with accession number DQ067898.1. To determine the performance of tobacco plants expressing PvPGIP2 under field conditions, trials were conducted in the tobacco area in Havana with a high inoculum pressure where *P. hyoscyami* f. sp. *tabacina* is a significant problem for tobacco production each year. During cold and wet season of 2009, the two transgenic Pv*pgip*2 lines and wild type plants were evaluated by planting fifty 8-week-old plants of each line in the tobacco production area. The plants were planted with a random design to look at positional effects in the field and five replicates were made. The percentages of healthy plants were determined, where more than three blue molds spot per leaf was considered as an unhealthy plant at 35 days post-planting. An arcsine transformation was performed on all percent incidence data before statistical analysis in order to improve homogeneity of variance. Data were subjected to analysis of variance or general linear model procedures of SAS (SAS Institute, Cary, NC, USA). Significant difference among means was determined by Fisher’s least significant difference mean separation at *P* = 0.05.

## RESULTS

**FIGURE 1:**
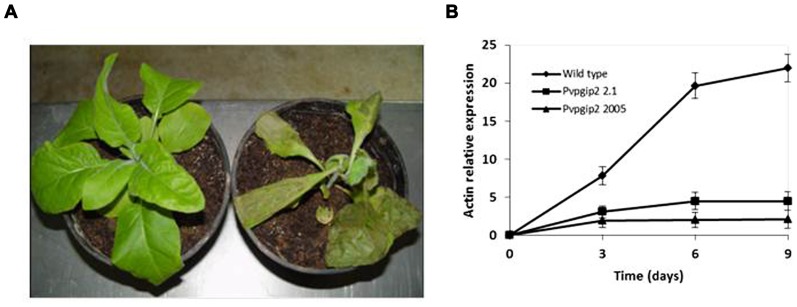
**Greenhouse evaluation of tobacco transgenic lines expressing the *Pvpgip2* gene inoculated with *R. solani***. Inoculated transgenic line Pvpgip2 2.1 **(A)** and wild type tobacco plants at 3 wpi. **(B)** Quantitative RT-PCR measuring *R. solani* growth in tobacco transgenic lines and wild type plants. Accumulation of fungal actin PCR transcripts was determined using tobacco 26S rRNA gene as a reference. Each point represents mean values with standard error (*N* = 5). The photographs were obtained at 50 cm of distance.

One of the genes of common bean (*Pvpgip2*) encodes the most efficient and wide-spectrum PG inhibitor so far studied (Casasoli et al., 2009). To assess the effectiveness of PvPGIP2 in protecting tobacco against *R. solani*, *P. parasitica* var. *nicotianae*, and *P. hyoscyami* f. sp*. tabacina*, two transgenic lines were evaluated. Under greenhouse conditions, the main symptoms caused by *R. solani* on wild type tobacco were small stem water-soaked lesions that rapidly become brown and sunken, primarily at the level of the soil line or closely above it. Lesions subsequently expanded throughout the stems causing the tissue to turn brown and die. Disease symptoms (both seedling death and stem rot) were severe on wild type plants and very limited and less visible in transgenic lines (**Table 1**). The two transgenic lines behaved similarly (line 2.1 is shown in **Figure 1A** as a representative result). Symptom development coincided with an increase of fungal biomass in the colonized roots of the wild type plants while no significant increase of fungal biomass occurred in transgenic plants (**Figure 1B**).

**Table 1:** Reaction of tobacco plants expressing *Pvpgip2* to *R. solani* in greenhouse conditions.

Genotype name	Disease incidence (%)^a^	
	Seedling death^b^	Stem rot
*Nicotiana tabacum *cv. SR1 expressing *Pvpgip2 *line 2.1	16.3*	14.2*
*Nicotiana tabacum *cv. SR1 expressing *Pvpgip2 *line 2005	15.8*	17.2*
*Nicotiana tabacum *cv. SR1	42.4^§^	52.8^§^
CV (%)^c^	6.2	7.1

a *Arcsine-transformed percentage of disease incidence*.

b *Seedling death was measured at 3 weeks post-inoculation*.

c *Coefficient of variation (N = 50). Values designated with the same symbol are not significantly different (P > 0.05)*.

**FIGURE 2:**
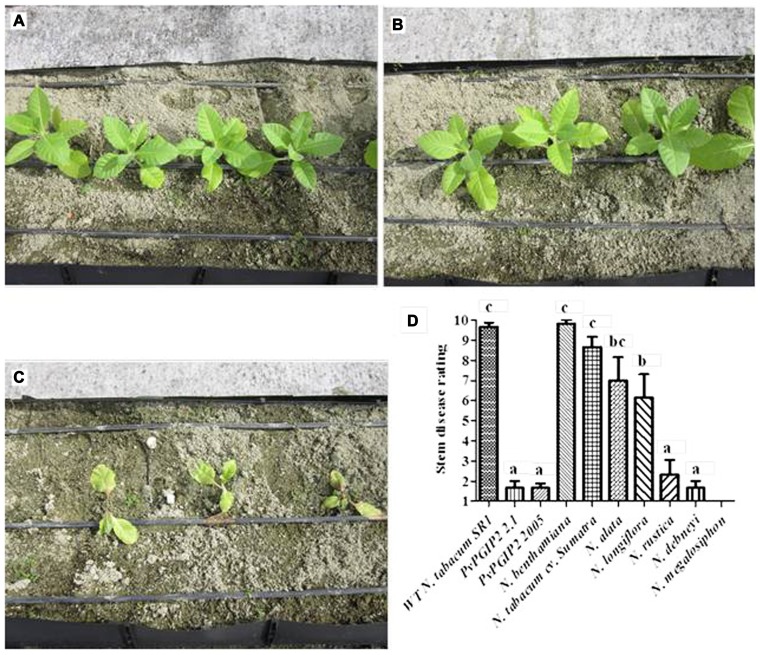
**Evaluation of *Pvpgip2* transgenic tobacco plants for resistance against *P. parasitica* var. *nicotianae***. Phenotype of transgenic tobacco lines Pvpgip2 2.1 **(A)**, Pvpgip2 2005 **(B)**, and wild type plants **(C)** on a highly infected soil at 10 days after planting. The development of stem lesions was evaluated using a linear scale of 1–10, where 1 was no disease and 10 was a dead plant according to Csinos (1999). Comparative evaluation of resistance in two transgenic homozygous lines expressing PvPGIP2 and reference genotypes of *Nicotiana* plants with different degree of resistance in greenhouse conditions **(D)**. Bars represent mean values with standard error (*N* = 50). Columns designated with the same letter are not significantly different (*P* > 0.05). The photographs were obtained at 100 cm of distance and are representative of one typical event. *Nicotiana rustica* (PI 499174), *Nicotiana alata* (PI 42334), *Nicotiana longiflora* (PI 555533), *Nicotiana benthamiana* (PI 555478), *Nicotiana debneyi* (PI 503320), *Nicotiana megalosiphon* (PI 555536).

Under greenhouse conditions the two transgenic tobacco lines expressing *Pvpgip2* were also remarkably resistant to the oomycete pathogen *P. parasitica* var. *nicotianae*. At 2-week post-inoculation slight disease symptoms appeared on the wild type plants whereas no symptoms were detected on the transgenic plants. However, at 5-week post-inoculation severe disease symptoms (leaf wilting and stem rot) were observed in the wild type plants. All wild type plants died 5 days later. Instead both transgenic lines expressing *Pvpgip2* remained healthy and showed a level of resistance similar to *Nicotiana* species that are naturally highly resistant to *P. parasitica* var. *nicotianae* (**Figures 2A–D**).

**FIGURE 3:**
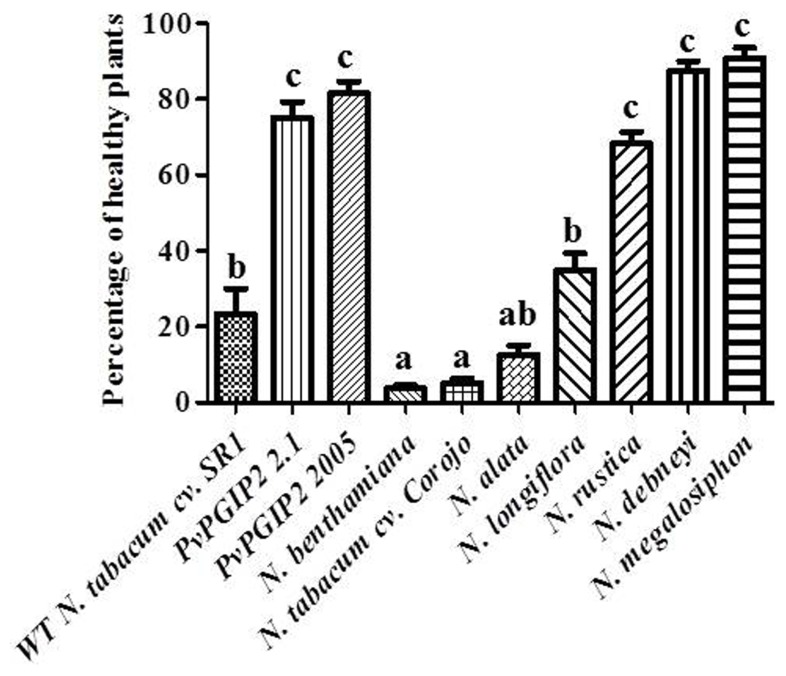
**Field trials of *Pvpgip2* transgenic tobacco plants for resistance against *P. hyoscyami* f. sp. *tabacina***. Quantitative evaluation of resistance to *P. hyoscyami *f. sp. *tabacina* in different transgenic lines and reference genotypes. *Nicotiana* plants exhibiting different degree of natural resistance at 35 days after planting. Bars represent mean values with standard error (*N* = 50). Columns designated with the same letter are not significantly different (*P* > 0.05).

Trials were also conducted in the field during the cold and wet season when tobacco blue mold caused by *P. hyoscyami* f. sp*. tabacina* constitutes a significant problem in Cuba. Transgenic plants displayed a high level of resistance that was comparable to that of *Nicotiana* species that are naturally highly resistant to *P. hyoscyami* f. sp. *tabacina* (**Figure 3**).

## DISCUSSION

We have shown that the expression of a *PGIP* gene from common bean in tobacco, i.e., a plant belonging to the economically important family of Solanaceae, confers to transgenic plants a strong resistance against fungi and oomycetes, both in greenhouses and in the field (*P. hyoscyami* f. sp*. tabacina*). Oomycetes are a group of eukaryotic microorganisms that includes some of the most important pathogens of plants. Among these, members of the genus *Phytophthora* cause enormous economic losses on crop species as well as environmental damages in natural ecosystems (Kamoun, 2006). PGs are produced by *Phytophthora* spp. and *R. solani* and are encoded by gene families (Marcus et al., 1986; Götesson et al., 2002; Wu et al., 2008). The PG–PGIP interaction has been characterized in *R. solani* (Akhgari et al., 2012) while many PG genes have been cloned from *Phytophthora* spp., but their interaction with PGIP2 of *P. vulgaris* has not yet been analyzed. However it is possible that the PG–PGIP interaction characterized *in vitro* does not reflect the situation *in vivo*. For example, as reported by Joubert et al. (2007), transgenic tobacco plants expressing grapevine PGIP are protected against *B. cinerea* without any evidence of PG–PGIPinteraction *in vitro*. It is possible that protection is due to effects other than the inhibition of pathogen PGs, caused by the overexpression of PGIP. PGIP may bind the most exposed and vulnerable positions in the pectin and indirectly protect pectin against degradation (Spadoni et al., 2006). On the other hand, it has been reported that transgenic plants expressing PGIP exhibit altered regulation of cell wall-associated genes; thus, the consequent cell wall modifications may be responsible for the enhanced resistance (Alexandersson et al., 2011).

The expression of a PG inhibitor neither alters the physiological performances nor exhibits detrimental effects on the growth of transgenic plants (Desiderio et al., 1997; Powell et al., 2000; Capodicasa et al., 2004; Manfredini et al., 2005; Alexandersson et al., 2011; Mohammadzadeh et al., 2012). It is therefore possible to engineer disease resistance in crop plants by using PGIPs as gene tools. The structure of PGIPs and of microbial PGs is being deeply studied; it is known, for example, that the change of one or a few residues confers to the inhibitor new recognition specificities and may improve its inhibitory strength (Leckie et al., 1999; Federici et al., 2001; Di Matteo et al., 2003; Casasoli et al., 2009; Benedetti et al., 2011). This knowledge may help in planning mutational strategies aimed at improving the properties of the natural PGIPs and their recognition versatility against the many microbial PGs evolved in nature.

## Conflict of Interest Statement

The authors declare that the research was conducted in the absence of any commercial or financial relationships that could be construed as a potential conflict of interest.
